# Cavernous hemangioma in unusual location: pterygopalatine fossa^☆^

**DOI:** 10.1016/j.bjorl.2016.02.003

**Published:** 2016-04-13

**Authors:** Bayram Şahin, Said Sönmez, Emine Dilek Yılmazbayhan, Kadir Serkan Orhan

**Affiliations:** a University of Istanbul, Istanbul Medical Faculty, Department of Otorhinolaryngology & Head and Neck Surgery, Istanbul, Turkey; b University of Istanbul, Istanbul Medical Faculty, Department of Pathology, Istanbul, Turkey

## Introduction

Hemangiomas are a benign vascular tumor and the most common benign tumors of the head and neck region. Although they usually present at birth or arise early in life, most of them involute spontaneously before adulthood. However, typically these tumors are located outside of paranasal sinuses and nasal cavity, including the lateral skull base, parotid gland, larynx, tongue and skin.^1^ Three major subtypes were described as capillary, cavernous and mixed. In the majority of the cases, the cavernous type is associated with the lateral wall of the nasal cavity or with the inferior turbinate.^2^, ^3^

In this article, we reported a hemangioma originating from the pterygopalatine fossa, treated via the endoscopic approach without any complication. To our knowledge, the cavernous hemangioma of pterygopalatine fossa has not previously been published in the English literature.

## Case report

**Figure 1:**
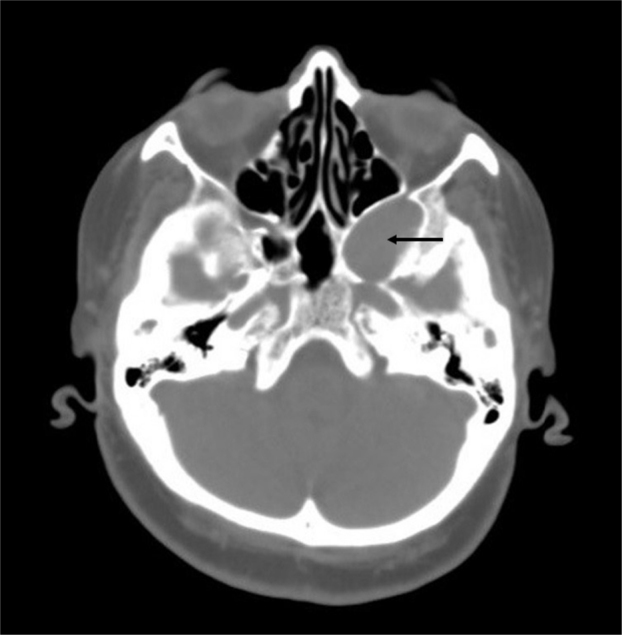
Preoperative axial CT scan. CT scan shows a soft tissue mass, which is located in the pterygopalatine fossa, causing expansion of the left cavernous sinus. Also, it pushes the posterior wall of the maxillary sinus toward the anterior, and the lateral wall of the sphenoid sinus toward the medial (arrow indicates tumor).

A 65-year-old female presented with headache and fullness on the face for several months. No other concomitant complaint was present. Nasal endoscopy, otological evaluation and cranial nerves examination were normal. Laboratory examination, complete blood count and routine blood chemistry were within the normal range. CT scan showed a well-circumscribed 3.4 cm × 2.8 cm soft tissue mass extending from the left pterygopalatine fossa to the left cavernous sinus (Fig. 1). It caused the remodeling in the posterolateral wall of the maxillary sinus, and the expansion at the cavernous sinus. Also, the mass is pushing the posterior wall of the maxillary sinus toward the anterior, and the lateral wall of the sphenoid sinus toward the medial.

**Figure 2:**
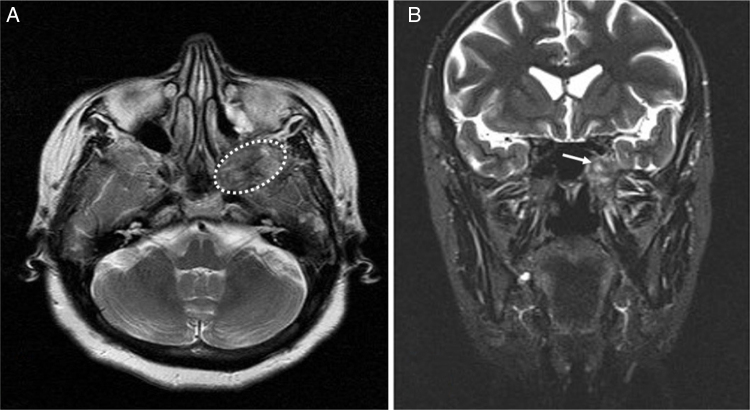
(A) Preoperative T1-weighted axial MRI. The mass is hypointense on the T1-weighted images (dashed-line indicates tumor). (B) Preoperative T2-weighted coronal MRI. The mass is mildly hyperintense on the T2-weighted images (arrow indicates tumor).

In MRI, dense-heterogeneous contrasted mass was detected and it was mildly hyperintense on the T2-weighted images and hypointense on the T1-weighted images (Fig. 2).

**Figure 3:**
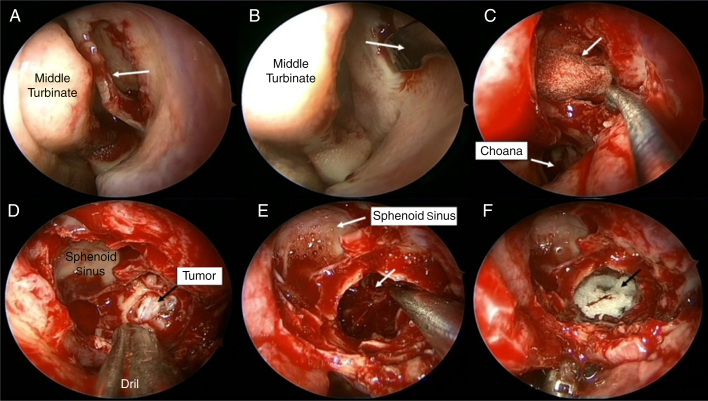
Intraoperative view. (A) Uncinate process was removed by backbiter forceps and later the ostium of the maxillary sinus was identified (arrow indicates uncinate process). (B) The medial wall of the maxillary sinus was removed and wide meatal antrostomy was made (arrow indicates maxillary sinus). (C) The anterior wall of the sphenoid sinus was removed partially and sphenoidotomy was made (arrow indicates nasal packing in the sphenoid sinus). (D) Posterior wall of the maxillary sinus was removed partially and the tumor was seen in pterygopalatine fossa. (E) Tumor was excised totally (arrow and aspirator indicate pterygopalatine fossa). (F) At the end of the surgery absorbable hemostat was filled into the pterygopalatine fossa (arrow indicates absorbable hemostat).

**Figure 4:**
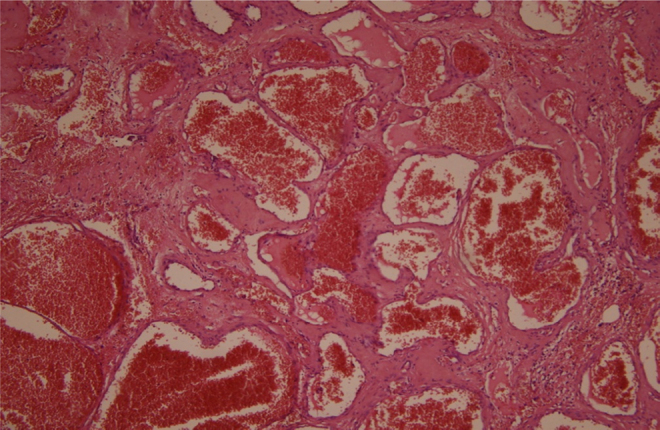
Histological examination. Branching vascular spaces consistent with hemangioma, H–E, 100×.

Endoscopic endonasal resection of the vascular lesion was performed under general anesthesia, without any complications. In operation, first, the medial wall of the maxillary sinus was removed and wide antrostomy was made. Anterior and posterior ethmoidectomy was added for increased visualization to posterior portion of the maxillary sinus. After that, sphenoidotomy was made and opticocarotid recess was identified for evaluation of the relationship with cavernous sinus. Posterior wall of the maxillary sinus was removed partially and the tumor was seen in PPF (Fig. 3). At this stage, sphenopalatine artery was cauterized against the possibility of bleeding. Finally, the tumor was dissected from near anatomical structures with bipolar cautery and excised totally. Histopathological examination was reported as cavernous hemangioma (Fig. 4).

## Discussion

Hemangiomas are benign vascular tumors most commonly found in the head and neck region, whereas these lesions rarely originate from the sinonasal area. They may arise from osseous, mucosal and submucosal portions of the nasal cavity and paranasal sinuses. In the literature, numerous classification systems were described for hemangiomas, with histological subtyping being the most commonly accepted. They are classified into three subtypes, according to dominant vessel size at the histological evaluation, as capillary, cavernous and mixed.^4^, ^5^

Capillary hemangiomas are the most common and are more often seen in children; they are usually located in oral mucosa, tongue and skin. These lesions usually may regress spontaneously, in early childhood; for this reason, surgical treatment is not recommended.^4^ Cavernous hemangiomas are uncommon congenital malformations and they may manifest during adulthood and do not involute spontaneously.^3^, ^6^

**Figure 5:**
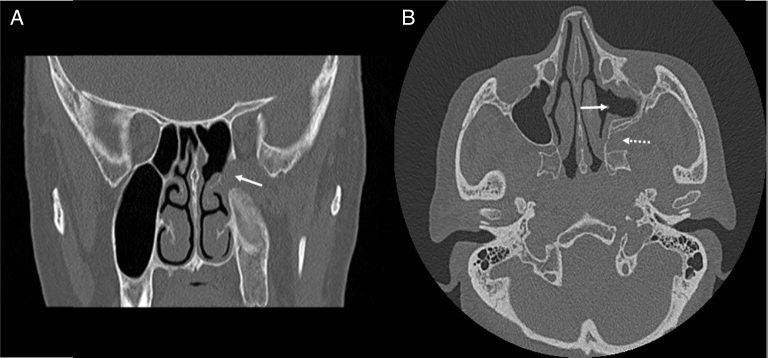
Postoperative CT images. (A) Coronal CT scan. There is no tumor recurrence in the pterygopalatine fossa (arrow indicates pterygopalatine fossa). (B) Axial CT scan. Left maxillary sinus and pterygopalatine fossa are seen normal (arrow indicates wide meatal antrostomy and dashed arrow indicates pterygopalatine fossa).

The surgical approach to Pterygopalatine Fossa (PPF) is difficult, for anatomical reasons (Fig. 5). The PPF is located anterior to the pterygoid plates, posterior to the maxillary sinus and inferior to the middle cranial fossa. PPF mostly includes lesions arising from skull base, nasal and oral cavity and orbit. In differential diagnosis, numerous diseases must be kept in mind for PPF masses, such as epidermoid cyst, meningoceles, carcinomas, melanomas, schwannomas, neurofibromas, neurofibrosarcomas, chordomas, and teratomas.^7^ Standard surgical approach to the PPF is Caldwell-Luc procedure. However, this technique offers a limited intraoperative view and requires gingivobuccal sulcus incision. Also, it is associated with some complications, such as recurrent sinusitis, pain or numbness on the face and teeth, facial swelling and infraorbital nerve and vessels injury.^8^, ^9^

In our case, fine-needle aspiration cytology was not performed, because the mass was located in an area which was not accessible with a fine needle aspiration biopsy. Only blood analysis, imaging modalities and clinical examination were used for diagnosis. At the preoperative radiological examination the mass was reported as a soft tissue tumor. The endoscopic transnasal approach was used for excision of a soft tissue tumor extending from the PPF to the cavernous sinus.

Preoperative CT and MRI are useful in defining the location and extension of hemangiomas. CT findings of the cavernous hemangiomas are a soft-tissue density circumscribed mass, and also enhancing after injection of contrast. Hemangiomas of nasal cavity and paranasal sinuses may cause some changes on the adjacent bone. Generally, these changes seem like benign on imaging modalities. Dillon et al. reported three of six patients with cavernous hemangioma of nasal cavity have adjacent bone changes. In addition, all three cases have benign changes consisting of remodeling and expansion.^5^ On the other hand, hemangiomas demonstrate an iso or hypointense signal on T1-weighted MRI and an hyperintense signal on T2-weighted imaging. Also, these tumors show intense contrast enhancement.^10^

Angiography and transarterial embolization are precious for the diagnosis and treatment of hemangiomas because biopsy or surgery may cause bleeding. Transarterial embolization may be used as an alternative treatment option for cavernous hemangiomas.

Suitable treatment of a sinonasal hemangioma is wide excision of the tumor with the underlying soft tissue or mucosa, and cauterisation or ligation of the feeding vessels. Preoperative embolization may be useful for extensive lesion.

## Conclusion

In conclusion, cavernous hemangiomas are rare, benign lesions of the nasal cavity and paranasal sinuses. The present case is the first well-documented primary cavernous hemangioma of the pterygopalatine fossa. We completely removed this lesion via the endoscopic transnasal approach without preoperative embolization.

## Conflicts of interest

The authors declare no conflicts of interest.
